# The Recent Progress on Nickel@Silver Metals Core@Shell Nanoparticles Application in Printed Conductive Materials – A Mini-Review

**DOI:** 10.2147/NSA.S509925

**Published:** 2025-04-14

**Authors:** Anna Pajor-Świerzy, Krzysztof Szczepanowicz

**Affiliations:** 1 Jerzy Haber Institute of Catalysis and Surface Chemistry Polish Academy of Sciences, Kraków, 30-239, Poland

**Keywords:** nickel@silver core@shell nanoparticles, conductive inks, sintering methods, conductive coatings, printed electronics

## Abstract

This mini-review paper gives a brief summary of recent works in the development of bimetallic core@shell nanoparticles composed of nickel (as a core) and a silver shell (Ni@Ag NPs). We present the methods of Ni@Ag NPs synthesis, ink preparation, and their coatings formation. We also place emphasis on the selection and optimization of the sintering process of materials based on Ni@Ag NPs. Finally, the challenges in the application of Ni@Ag NPs in printed conductive structures are presented.

## Introduction

The fabrication process of high-performance with large area and low-cost printed electronics requires a combination of well-defined conductive structures, lowering materials usage, and high resolution.^1–4^ To avoid printing defects including undesired discontinuities and lack of circuit connections, which can lower the efficiency of the printed electronics, the composition of inks as well as printing conditions have to be controlled and optimized. Moreover, critical parameters for obtaining electronic devices with high-performance are the structural and conductive properties of printed patterns, adhesion between metallic circuits (or coatings), and substrate.

The first step in the manufacture of printed conductive materials is the selection of the proper components of inks or pastes to provide high quality and performance. From this point of view, the conductive ink (paste) is composed of active material (eg metallic NPs, carbon nanotubes, graphene sheets), various types of vehicles in an organic or aqueous form, and additives (eg wetting agents, defoamers, humectants).^1–4^ The most important component of such ink is the conductive material, usually in the form of metallic nanoparticles due to their low temperature of melting and good conductive properties. Their preparation with optimal physiochemical properties, eg size, conductivity, or stability against the aggregation process is one of the main factors required for the preparation of conductive structures with high-performance properties.

The top metals in terms of conductive applications are silver and gold.^5^,^6^ Although conductive nanomaterials based on Au and Ag NPs have good conductive properties as well as stability against the oxidation process, they are costly materials. Therefore, there are many other efforts to prepare conductive inks or pastes from cheaper materials of non-noble metals such as copper or nickel.^7^,^8^ However, electronic circuits or conductive coatings composed of Cu NPs have low mechanical strength. Therefore, their application in the fabrication process of electronics can be limited, while the ones based on nickel show better resistance against destruction or plastic deformation.^9^ The tendency of Cu and Ni NPs to oxidation process in an atmospheric environment is a fundamental problem due to lowering or even definitively loss of the conductivity of the printed structures, which can limit their application. To protect Cu or Ni nanoparticles from this undesirable oxidation process, various approaches have been proposed so far. One of them was the deposition on their surface of a protective layer of alkanethiols, polymers, surfactants, or long-chain carboxylic acids as capping agents.^10^,^11^ However, this approach only delays the oxidation of NPs and does not provide their long-term stability against this process. Taking into account this point of view, the best method to provide good stability of Cu and Ni NPs against the oxidation process is coating them with noble metals. Formation of such a protective shell results in the obtaining of core-shell NPs, which can be synthesized by various types of methods such as electro or vacuum deposition as well as transmetallation reaction.^12^–^14^ The electrodeposition and vacuum processes require large financial outlays. Besides, the preparation of core-shell structures using such methods takes a lot of time. The transmetallation reaction (a galvanic displacement process) is a simple and low-cost wet chemical method, in which on the surface of the core particle the second metal with higher redox potential is reduced. The experimental as well as theoretical studies on the controlling of the thickness shell of the bimetallic core-shell nanoparticles can provide new ways for the preparation of a range of attractive structures with unique and specific properties. Taking into account this point of view, Wali et al^15^ modified the thickness of the gold shell of silver-gold core-shell NPs. They noticed an improvement in the photovoltaic properties of solar cells based on nanoparticles with thicker shells. The sintering mechanism of Cu@Ag core@shell NPs formed from the silver shell with various thicknesses for flexible conductive film preparation was analyzed by Zhang et al.^16^ They noticed that the resistivities of films based on Cu@Ag NPs with 5 nm of Ag shell decrease with increasing sintering temperature (140–220°C) from 248.66 to 54.75 μΩ∙cm. In contrast to the resistivity of coatings based on nanoparticles with thicker Ag shells of 10 nm. The lowering of resistivity from 110.37 μΩ∙cm to 28.36 μΩ∙cm was observed. Wen et al^17^ performed theoretical studies on the thermal stability of the Al@Pd core@shell NPs with different shell thicknesses. They observed more thermally stable nanoparticles with the thicker Pd shell. Moreover, the produced adiabatic temperature was higher by using these nanoparticles as a result of the greater content of Pd. The atomistic sintering simulation method was used for the investigation of the dependence of the sintering process Cu@Ag core-shell NPs on silver shell thickness. It was found that during sintering the plastic deformation process is prevented by thinner silver shells and the ratio of amorphous silver atoms between nanoparticles necks increases with increasing Ag shell thickness.^18^

The fabrication process of metallic circuits or coatings from inks (pastes) composed of core-shell NPs can be performed by a broad range of techniques such as roller pen,^19^ spin or bar coating,^20–22^ inkjet,^13^ or screen printing.^23^ The printing or coating process is only the first stage in the preparation of coatings or patterns with conductive properties. After the ink deposition, the direct contacts between metallic nanoparticles, within the ink coating on a substrate, have to be formed to obtain electrical patterns with high conductivity. Transformation of nonconductive ink coating to conductive one can be achieved by its exposure to intense light pulse, heat, plasma, microwave or UV irradiation, high electrical field, and contact with chemical reagents as sintering agents.^24–26^ Among various sintering techniques, the thermal process performed by heating the deposited metallic inks is still the most commonly used method. Grouchko et al^13^ sintered by heating the inkjet-printed patterns based on Cu@Ag NPs. They observed the decrease in the resistivity with the increasing sintering temperature. The obtained resistivity after heating at 300°C was only 7 times greater than that of bulk copper. As an alternative method for thermal sintering of Cu@Ag NPs, laser process was applied.^27^ In the optimized conditions, the formed in air layer showed an electrical resistivity of 28.5 μΩ·cm. Dai et al^28^ showed the “chemical” sintering method of Cu@Ag NPs by using NaBH_4_ as the chemical sintering agent. In this approach, they obtained a conductive structure at room temperature.

The long-term and thermal stability of the printed conductive materials based on bimetallic core@shell NPs is important for their industrial application. The high-conductive printed traces composed of Cu@Ni NPs with ultrahigh oxidation resistance even after heating at 250°C in the air were obtained by Li et al.^29^ Wang et al^30^ noticed that sintered films based on copper core@shell NPs, Cu(0)@Cu(I) containing a surface passivation layer of formate ions-involved Cu(I) coordination polymers, remained its original resistance (4.50 µΩ·cm) after 4 months of storage in the air at 5°C.

An exponential growth of interest in the application of core-shell bimetallic nanoparticles can be related to their release into the environment and impact on human health. It was found that metallic NPs can be toxic and lead to many diseases such as lung inflammation or heart disease.^31^ However, Długosz et al^32^ noticed that bimetallic nanoparticles of Ag@Cu and Cu@Ag exhibited no genotoxic properties, which makes them safer to use while maintaining antimicrobial properties. Besides, Abed et al^33^ analyzed the effect of Ag@Au core@shell NPs on human blood. They did not observe noticeable changes in the blood components, therefore they found out that such nanoparticles have to be non-toxic. Core-shell bimetallic nanoparticles can pass into the environment during the preparation process of materials based on them, their application, or following the disposal of products containing nanoparticles. The toxicity assessments of bimetallic Ag@Au NPs were investigated with *D. magna*, a freshwater filter-feeding crustacea by Li et al.^34^ Based on the experimental results, they concluded that the tested nanoparticles have shown dose-dependent ecotoxicological effects on *D. magna*. Unfortunately, the impact of Ni@Ag core@shell nanoparticles on human health as well as their intentional and unintentional disposal in the environment are largely unknown, and additional research is required.

In the recently published literature, the preparation methods of various types of bimetallic core@shell NPs (Ni@Pt, Ag@Pt, Ru@Pt, Cu@Ag, Au@Pd, Pd@Ag, Pd@Pt, Ag@Au, Au@Ag, Co@Pt) have been proposed.^13^,^35–39^

**Figure 1:**
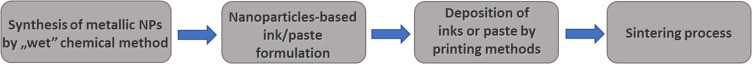
Schematic diagram of the fabrication process of materials with conductive properties.

In the presented review, we would like to focus on research on the synthesis and application of Ni@Ag NPs in the fabrication of printed coatings and patterns with conductive properties. Generally, to fabricate conductive patterns/coatings when using metallic nanoparticles few steps are important and required: (1) synthesis of metallic NPs; (2) preparation of nanoparticles-based ink or paste; (3) their deposition by printing or coating methods; (4) sintering process, as schematically presented in Figure 1.

From this point of view, our paper is focused on the various approaches to their preparation, stabilization against the oxidation process, formation of conductive materials (inks or pastes), coatings/patterns fabrication, and methods of the sintering process.

### Synthesis of Ni@Ag NPs

Generally, the metallic core@shell nanoparticles can be synthesized by chemical,^13^,^16^,^21^,^22^ physical,^20^ or by combination (chemical and physical) of those methods.^40^,^41^ However, methods for the synthesis of Ni@Ag NPs are mainly based on a chemical approach or physical approach. Chemical methods can be classified into two types: (1) “one-pot” (co-reduction) process, in which core@shell nanoparticles are formed in one-step reaction; (2) synthesis process is based on two-stages: (2a) preparation of the core, and (2b) shell formation by transmetalation (galvanic displacement) reaction.

In the synthesis process of core@shell NPs, the right selection of the reagents is crucial of importance. Therefore, the effect of various types of metal precursors, reducing agents as well as stabilizers, on the properties (size, shape, stability against aggregation process) of nickel NPs is important for their effective formation. Those NPs were synthesized in a liquid medium (“wet chemistry” method) by reduction of ions of nanoparticles precursor, with a proper reducing agent. The reduction of nickel ions to atoms takes place only if the reducing agent shows more negative redox potential than the metal nanoparticles precursor. To be of practical importance, the difference in reduction potential between the reducing agent and nanoparticles precursor should be larger than 0.3 V; in other ways, the progress of the reaction may be too slow or even the formation of nanoparticles may not occur.

**Figure 2:**
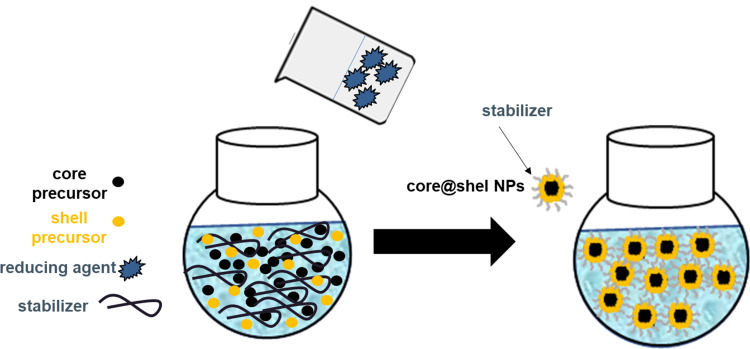
Scheme of the “one-pot” synthesis of bimetallic core@shell nanoparticles.

In the “one-pot” synthesis process of bimetallic core@shell nanoparticles, precursors of core and shell can be reduced simultaneously by a proper reducing agent as presented schematically in Figure 2.

**Figure 3:**
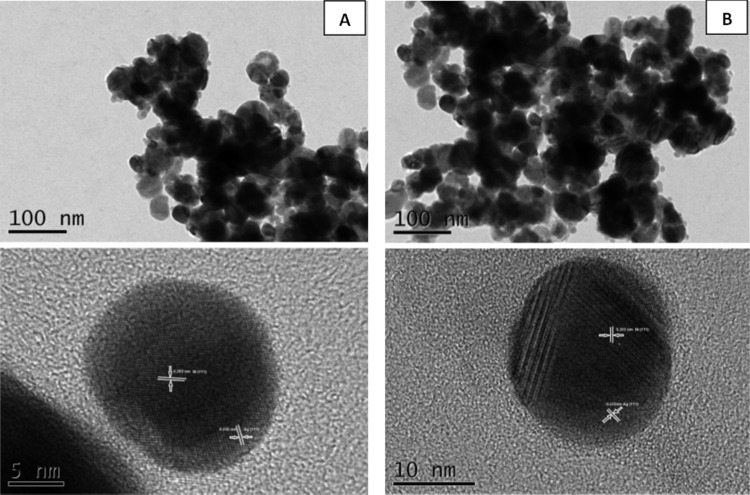
TEM images of Ni@Ag nanoparticles obtained by using polyol (**A**) and microemulsion methods (**B**). Reprinted fromThu NNA, Park JG, Kim S-H. Synthesis of Ni-Ag core-shell nanoparticles by polyol process and microemulsion process. Bull Korean Chem Soc. 2013;34:2865–2870. Copyright 2013 Korean Chemical Society.^45^

In one of the procedures of the co-reduction simultaneous process, Ni@Ag nanoparticles were prepared by the successive reduction (in ethylene glycol) of nickel chloride (as a precursor of core) and silver nitrate (as a precursor of shell) with hydrazine in the presence of cationic polymer (polyethyleneimine, PEI) as the protective agent.^42^ The monodisperse Ni@Ag NPs were synthesized. The diameter of the Ni core was 6.2 nm, and the thickness of the Ag shell was 0.85 nm. Moreover, the silver shell fully covered the nickel core. In the work of Chen et al,^43^ the synthesis process of nickel@silver NPs without using protective agents was proposed. They noticed that with increasing silver nitrate concentration the size of Ni@Ag NPs increased, indicating that nanoparticles with thicker shells were formed as well as the possibilities of the controlling of silver shell thickness. The Ni@Ag NPs at the size of 21 nm, in the synthesis process without a capping agent, were also obtained by Arasu et al.^44^ Thu et al^45^ for the Ni@Ag NPs synthesis used two methods: the polyol process and the water-in-oil microemulsion method. The polyol approach involved the use of as a reducing agent an organic solvent (ethylene glycol) for Ag ions with polymer (poly(vinyl-pyrrolidone), PVP) as a capping agent. The emulsion of water/cetyltrimethylammonium bromide (CTAB)/cyclohexane was prepared. In the polyol method, the nickel@silver core@shell NPs with 3 nm of silver shell deposited on 13 nm of nickel core were synthesized, while Ni core at the size of 8 nm coated with Ag shell with thickness of 6 nm after the microemulsion process was obtained, as is presented in Figure 3A and B, respectively. To investigate the stability against the thermal oxidation of Ni@Ag NPs synthesized by polyol and microemulsion techniques, they were heated at different temperatures. After this process, the changes in nanoparticles structure and phase were studied using the XRD method. The XRD pattern of the Ni@Ag nanoparticles showed only the presence of Ni and Ag phase until 300°C of heating and any oxides were observed. The obtained results indicate that the silver shell protects nickel core nanoparticles against oxidation.

The co-reduction synthesis process was also used for the fabrication of Ni@Ag NPs in the presence of WO_3_ nanorods for photocatalytic hydrogen detection.^46^ The lattice fringe of Ni@Ag/WO_3_ measured by HRTEM was 0.374 nm, while the TEM-EDX analysis shows that nickel and silver are present on the surface of WO_3_. The oxidation states of the elements in Ni@Ag/WO_3_ were studied by the XPS method. The obtained results confirm the presence of Ag and Ni without NiO, which indicates the formation of stable against oxidation nanoparticles.

One-pot approaches have some advantages and some disadvantages. The simplicity and cost can be included as advantages, while final purity is problematic. It is related to synthesis process conditions. The reaction dispersion contains a reducing agent, which can inhibit the complete reduction of silver ions in the shell. In this context, instead of shell, the Ag NPs will be formed or/and only some part of nickel will be coated with shell, a mixture of Ag NPs and Ni@Ag core@shell NPs will be present in the reaction dispersion. This phenomenon is the result of electron transfer from the reducing agent to the silver ions instead of the transfer process from the core NPs surface atoms.

**Figure 4:**
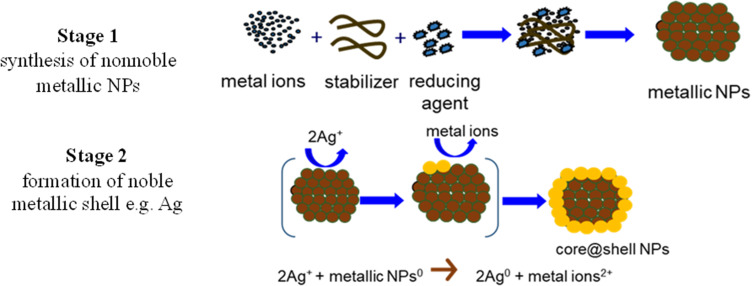
Scheme of the synthesis of bimetallic core@shell nanoparticles by using a two-step process.

The second, two-stage, approach of synthesis of metallic core@shell NPs based on the transmetalation (galvanic displacement) process is especially effective in the fabrication process of Ni@Ag nanoparticles according to a large difference between the redox potentials of non-noble Ni metal and noble Ag metal. Generally, the methodology of the formation of metallic nanoparticles (core), which are covered with noble metal layers (shell) to protect them from oxidation by using the transmetalation process, represents the two-step procedure illustrated in the scheme presented in Figure 4.

At the first stage of the synthesis process of Ni@Ag NPs, aqueous dispersions of Ni NPs as the core of bimetallic structure were prepared by the chemical reduction method. Among the reduction agents for the preparation of Ni cores, the most often the strong ones were used such as sodium borohydride^21^,^22^,^47–53^ and hydrazine.^42^,^52^,^54–56^

To improve the reduction process of Ni ions to nanoparticles, additionally, two types of complexing agents: AMP (aminomethyl propanol) and CA (citric acid) were applied.^48–52^

In the presented studies, the nickel NPs were synthesized using various types of their precursors. As the source of Ni ions their salt, for example, nickel acetate,^21^,^47^,^48^ nickel sulfate,^22^,^48–52^ or nickel nitrate,^53^ were used. An important factor determining the application of metallic NPs in conductive materials fabrication is their protection from aggregation and finally the precipitation process. To obtain stable Ni NPs regarding those phenomena, a proper stabilizer should be selected, and its concentration is also an important parameter since this component affects the lifetime and the performance of printed conductive inks or pastes. In this context, the colloidal stability of dispersions of Ni NPs can be optimized by using their synthesis process and various types of stabilizers. Among them, the polymers such as poly(acrylic acid),^21^,^47^ poly(sodium 4-styrene sulfonate),^47^ polyethylenimine,^42^,^47^ poly(N-vinyl-2-pyrrolidone),^47^ sodium carboxymethyl cellulose^22^,^48–52^ or polyacrilonitrile;^54^ organic acid (citric acid)^55^ or cationic surfactants such as cetyl trimethyl ammonium bromide^54^ or anionic sodium dodecyl sulphate^55^ were applied.

The presence of the non-consumed reducing agent in the dispersion of the synthesized Ni core nanoparticles is an important challenge when using the transmetalation process for preparation of Ni@Ag NPs. To solve this problem, several approaches were applied: (1) using stoichiometric concentration or a minor deficiency of the reducing agent relative to the core nanoparticles precursor;^47–52^ (2) thorough washing of the synthesized core nanoparticles by sequential rinsing-centrifugation process prior shell formation.^21^,^22^,^54^,^55^

To obtain stability against the oxidation of Ni NPs, the process of the deposition of the Ag shell by using transmetalation reaction has to be also optimized, by using the proper type as well as the amount of the precursor of the silver shell. As the source of silver ions in the synthesis of Ni@Ag NPs Ag-ammonia complex,^20^,^21^ AgNO_3_,^42^,^48–52^,^55^ Ag_2_SO_4_,^53^ or Tollens’ reagent^54^ have been used so far.

**Figure 5:**
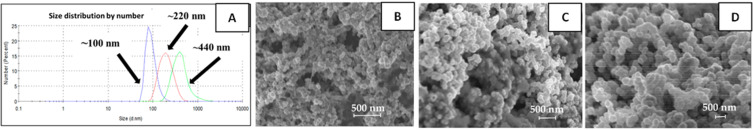
The characteristics of synthesized Ni@Ag NPs by transmetallation process: (**A**) various size distributions; SEM images of such NPs at the size of about 100 (**B**), 220 (**C**), and 440 (**D**) nm. Reprinted from Pajor-Świerzy A, Staśko D, Pawłowski R, Mordarski G, Kamyshny A, Szczepanowicz K. Polydispersity vs. monodispersity. How the properties of Ni-Ag core-shell nanoparticles affect the conductivity of ink coatings. Materials. 2021;14:2304. © 2021 by the authors. Licensee MDPI, Basel, Switzerland. This article is an open access article distributed under the terms and conditions of the Creative Commons Attribution (CC BY) license.^48^

By playing with a concentration of complexing agents (citric acid and aminomethyl propanol) and as the effect of the transmetalation process, the nickel@silver nanoparticles at various size distributions (of about 100, 220, and 420 nm) have been obtained,^48^ as presented in DLS analysis (Figure 5A) and SEM images (Figure 5B–D). The stability of Ni@Ag NPs with thickness shells of 20, 35, and 45 nm against the aggregation process was also analyzed.^53^ It was noticed that the size of obtained NPs almost did not change after 30 days of storage, which indicates their long-term stability regarding aggregation. Moreover, what was the most important, such NPs were characterized by good stability against oxidation due to the high atomic percent of metallic nickel (84%), which was confirmed by analysis of the chemical composition of the obtained Ni@Ag NPs dispersion.

**Figure 6:**
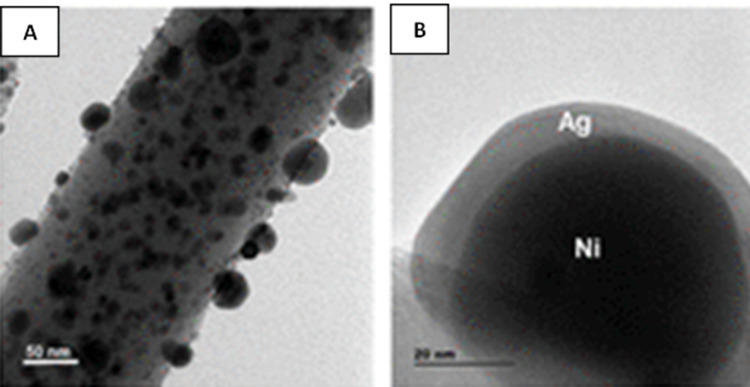
Representative TEM (**A**) and HRTEM (**B**) images of Ni@Ag NPs. Reprinted from Sunil N, Unnathpadi R, Pullithadathi B. Label-free SERS salivary biosensor based on Ni@Ag core−©shell nanoparticles anchored on carbon 440 nanofibers for prediagnosis of lung cancer. ACS Appl Nano Mater. 2023;6:11334–11350. Copyright © 2023 American Chemical Society.^52^

Sunil et al^52^ developed a synthesis process of bimetallic Ni@Ag NPs, via a two-step method, based on a chemical reduction of nickel chloride by hydrazine, followed by a transmetalation reaction, which was anchored on the carbon nanofibers (CNFs). They used high-resolution transmission electron microscopy (HRTEM) to determine the morphology and microstructural properties of the obtained nanoparticles. As can be seen in Figure 6A, they obtained Ni@Ag nanoparticles with a homogeneous distribution and good dispersion. Moreover, it can be seen that such NPs were strongly adhered to the surface of the CNFs. Figure 6B presents that a silver shell (lighter contrast) was successfully deposited on the nickel core (darker contrast), which proved the formation of the Ni@Ag core@shell structure.

**Figure 7:**
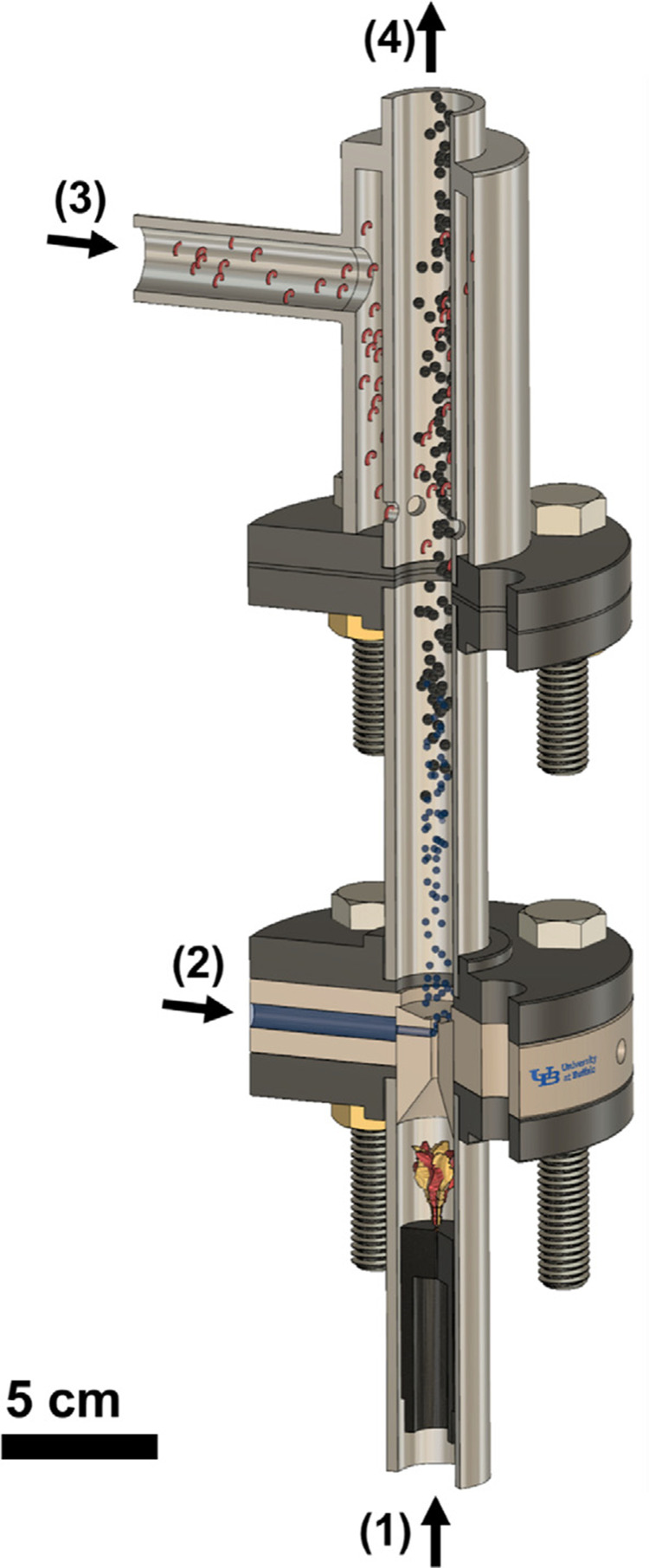
A scheme of the HTRJ reactor and synthesis process of Ni@Ag NPs flame-driven reaction. Reprinted from Mohammadi MM, Gunturi SS, Shao S, Konda S. Flame-synthesized nickel-silver nanoparticle inks provide high conductivity without sintering. 450 Chem Eng J. 2019;372:648–655. © 2019 Elsevier B.V. All rights reserved.^57^

The physical method of nanopowders synthesis of Ni@Ag by using a flame-driven High-Temperature Reducing Jet (HTRJ) process was demonstrated by Mohammadi et al.^57^ A schema of the HTRJ reactor for the synthesis of Ni@Ag NPs is shown in Figure 7. Such process involved a few stages: (1) formation of an inverted diffusion flame by using oxygen, hydrogen, and nitrogen as oxidant, fuel, and carrier gas, respectively; (2) injection of metal nitrate aqueous precursor (which after mixing process with the combustion products with high-velocity is atomized, evaporated, and finally decomposed) and formation of nanoparticles in a reducing environment, followed by their nucleation and growing in the reaction chamber; (3) mixing of obtained Ni@Ag NPs with vapor of octylamine; (4) exit of the functionalized nanoparticles from quench zone for downstream collection. As a result of this reaction, core@shell NPs at the diameter of 4–5 nm were synthesized. Besides, XRD analysis confirmed the stability of obtained nanoparticles against oxidation. In XRD patterns, any nickel oxide peaks were observed after the addition of silver shell precursor.

### Conductive Coatings Fabrication

The conductive nanomaterial (eg Ni@Ag NPs) is the most important component of nanoink for conductive patterns or coatings preparation. Generally, the coatings or printed patterns based on ink with high metallic NPs loading show higher conductivity since the more concentrated nanoparticles dispersion forms a higher number of necks and percolation paths between nanoparticles in the deposited films or printed circuits. Therefore, the nanoparticles loading in ink dispersion should be in the range of 20–80 wt%.^58^

The inks based on Ni@Ag NPs with a concentration of 15–25%,^21^,^22^,^47–51^ or 60%^55^ have been fabricated so far. The process of nanoinks formation involves a few steps: (1) removing an excess of organic materials (such as stabilizer) by the washing of Ni@Ag NPs with a proper solvent such as water,^21^,^22^,^48–51^ ethanol^55^,^56^ or glycerol;^21^ (2) concentration process by centrifugation^21^,^22^,^48–51^,^55^,^56^ or their collection in the chamber;^57^ (3) their redispersion in water,^21^,^22^,^48–51^ or organic vehicles;^55–57^ (4) optimization of ink properties by using modifiers of surface tension and rheological properties, defoamers, binders, or humectants, that provide conductive patterns or coatings preparation with optimal conditions^1^,^2^,^9^,^42^ (for example, by using the wetting agent of BYK 348,^47^ Surfynol PSA 336,^22^,^47^,^49^ or TEGO WET KL 245^21^,^46^ the coatings with high-quality were fabricated) (5) obtaining of the homogenous dispersion of ink formulation composed of Ni@Ag NPs and required additives by ultrasonication,^21^,^22^,^46–49^ ball milling,^53^ or mild sonication.^56^

The selection of the proper solvent, as well as ink additives, is important for the ink properties and also its application in electronics. The adjustment of viscosity and wettability of the ink formulation allows for obtaining materials that show high conductivity. Inks based on organic solvent^21^ have the main advantages of application in conductive materials such as low-viscosity and fast-drying. Therefore, they usually have better processability than water-based inks. However, the high cost and toxicity of organic solvents limit their use in the large-scale fabrication of inks based on them. Besides, water is an environmentally friendly solvent and has a low-cost and nonflammable nature. An attractive feature of water as a solvent was the motivation to produce water-based conductive inks.^21^,^22^,^47–51^ However, it is not always possible to apply water-based ink to obtain conductive materials. The type of solvent and additives of ink should be adjusted to the printing method. For example, for the screen printing method, the viscosity of ink was increased by changing the solvent from water to glycerol.^21^ Moreover, PVP polymer was added at the optimal concentration of 3% to the ink formulation to increase viscosity as well as its wettability. Mohammadi et al^57^ prepared stable and uniform suspensions of nano-inks in non-polar solvents such as toluene.

The conductive patterns based on Ni@Ag NPs are usually fabricated by using two steps: (1) the deposition of inks by proper coating or printing methods, and (2) the sintering process, in which nonconductive materials are transformed into conductive ones.

**Figure 8:**
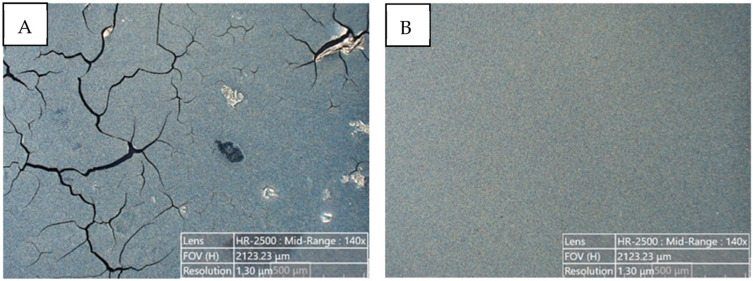
Examples of optical microscopy images of coatings, deposited by a bar coating method, composed of Ni@Ag nanoparticles, dried at 80 °C for 15 min: (**A**) without a wetting agent; (**B**) with BYK 348 at optimal concentration (0.05%). Reprinted from Pajor-Świerzy A, Szyk-Warszyńska L, Duraczyńska D, Szczepanowicz K. UV-Vis sintering process for fabrication of conductive coatings based on Ni-Ag core–shell nanoparticles. Materials. 2023;16:7218. © 2023 by the authors. Licensee MDPI, Basel, Switzerland. This article is an open access article distributed under the terms and conditions of the Creative Commons Attribution (CC BY) license.^50^

As the coatings methods of inks composed of Ni@Ag NPs screen printing,^21^,^53^ bar coating,^22^,^46–49^ or spin-coating^56^ were used. It was observed, that the ink composition has to be adjusted to the method of their deposition to obtain coatings with good quality, which requires a proper wetting agent at optimal concentration. For example, as is presented in Figure 8A, the coating based on Ni@Ag NPs, deposited by a bar coating method, without a wetting agent was nonuniform, and some holes and cracks were observed, while the coating formed with the addition of 0.05% of BYK 348 showed good quality, as can be seen in Figure 8B.^48^

After the deposition or printing process of metallic inks based on Ni@Ag NPs for obtaining the coatings or patterns with low resistivity, eg conductivity close to that of the bulk metal, their additional treatment (sintering) is required. Sintering results in desorption and/or decomposition of the insulating organic materials surrounding nanoparticles, which provides neck formation between them, and grain growth is observed. As a result of this phenomenon tightly packed or welded dense continuous metallic films with high conductivity are formed.^1–4^,^58–60^

Thermal sintering of the deposited coatings or patterns is still the most commonly used sintering technique. A few factors affect the final conductive properties of materials based on Ni@Ag NPs sintered by a thermal process such as the applied temperature, sintering time, size of nanoparticles, silver shell thickness, as well as the type and concentration of organic additives (eg polymeric stabilizers). In this context, the effect of the size of Ni@Ag NPs as well as sintering temperature and time on the conductivity of coatings formed from them was investigated.^21^ Such films prepared from inks based on Ni@Ag NPs at the average size of 70 and 250 nm were sintered at the temperature range 250–370°C and times of 10–60 min. It was noticed that among the coatings based on Ni@Ag NPs with a size of 70 or 250 nm, the lower value of sheet resistance (0.254±0.05 Ω/) can be achieved for the films composed of Ni@Ag NPs at the average size of 250 nm after thermal sintering performed at 350°C for 30 min. The obtained resistivity of the coating with a thickness of about 2 µm was 63 μΩ·cm (11% conductivity of that for bulk nickel). It suggests that those conditions of the sintering process allowed for removing the organic components without oxidizing NPs, which resulted in the formation of an interparticle connection. Additionally, in the presented research,^21^ the dependence of the conductive properties of ink coatings based on the mixture of Ni@Ag NPs of two sizes 70 and 250 nm on their concentration was studied. The value of resistivity of films composed of those nanoparticles deposited by using two different methods (bar coating and screen printing) was compared. A significant decrease in the resistivity of coatings composed of a mixture of Ni@Ag NPs at the size 70 and 250 nm (at mass ratio 1:1) was noticed after sintering at 300°C. The obtained conductivity was about 20% of that for a bulk nickel. This result can be explained by the formation of more compact conductive tracks in the sintered coatings as the result of a larger number of contact points between nanoparticles with different size distributions.

**Figure 9:**
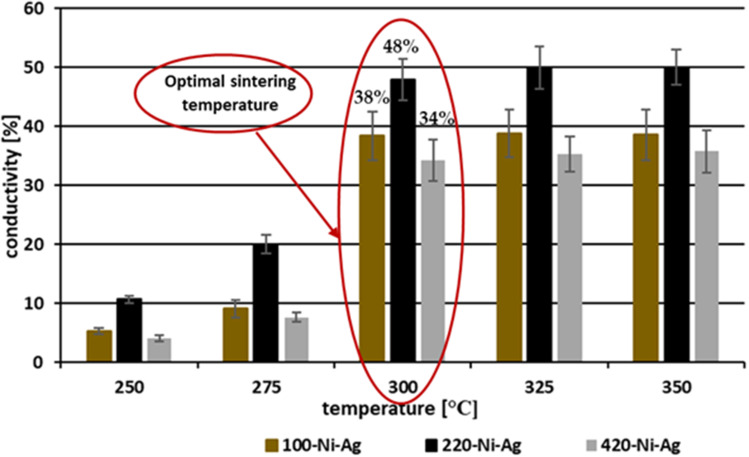
The effect of the size of Ni@Ag NPs on the conductivity (in comparison to that for bulk nickel) of metallic coatings sintered at the temperature range from 250–350 °C (30 min). Reprinted from Pajor-Świerzy A, Staśko D, Pawłowski R, Mordarski G, Kamyshny A, Szczepanowicz K. Polydispersity vs. monodispersity. How the properties of Ni-Ag core-shell nanoparticles affect the conductivity of ink coatings. Materials. 2021;14:2304. © 2021 by the authors. Licensee MDPI, Basel, Switzerland. This article is an open access article distributed under the terms and conditions of the Creative Commons Attribution (CC BY) license.^48^

The thermal approach of the sintering process for the preparation of conductive coatings formed from Ni@Ag nanoparticles at average sizes of 100, 220, and 420 nm was also applied.^48^ The coated films were sintered at the temperature range from 250°C to 350°C. Firstly, the dependence of their conductive properties on the size of individual nickel@silver core@shell NPs was investigated. As can be seen in Figure 9, the coatings composed of nanoparticles at an average size of about 220 nm show the highest conductivity at applied sintering temperature from 250°C to 350°C, which suggests that the size of nanoparticles affects the conductivity of sintered films. It can be also noticed that the optimal sintering temperature for coatings formed from all types of Ni@Ag NPs was 300°C. The use of higher temperatures for heating almost did not affect the conductivity of such films. At the optimal sintering conditions (300°C and 30 min), coatings composed of Ni@Ag NPs at the average size of about 220 nm have the highest conductivity corresponding to 48% of that for bulk nickel.

It also analyzed the impact of the polydispersity of Ni@Ag nanoparticles on the final conductivity of coatings based on them.^48^ For this purpose, dispersions of nanoparticles at various sizes (100, 220, 420 nm) were mixed, and the coatings created from them were subjected to the sintering process under optimal conditions (300°C, 30 min). It was observed that coatings composed of a mixture of NPs at a weight ratio of 1.0:1.5:0.5 with average sizes of 110, 220, and 420 show the highest increase in conductivity. The obtained resistivities and conductivity were 10 μΩ·cm and 69% of the bulk nickel, respectively. The improvement of the conductive properties of coatings composed of the mixture of Ni@Ag NPs at different size distributions can be explained as a result of the better packing in sintered films. The voids between larger particles are filled with smaller ones providing the formation of more compact coatings and enabling an effective merging of NPs during the sintering process.

The effect of the silver shell thickness of Ni@Ag core@shell nanoparticles on the process of preparation and sintering of conductive coatings obtained by the thermal process was studied by Pajor-Świerzy et al.^51^ The higher conductivity for the films composed of Ni@Ag NPs with the thicker silver shell was obtained. It was also noticed that the thickness of Ag shells affects the sintering temperature of coatings. A lowering of temperature for films based on nickel@silver NPs with thicker shells due to a lower melting point and a higher conductivity of silver in comparison to nickel nanoparticles was achieved.

**Figure 10:**
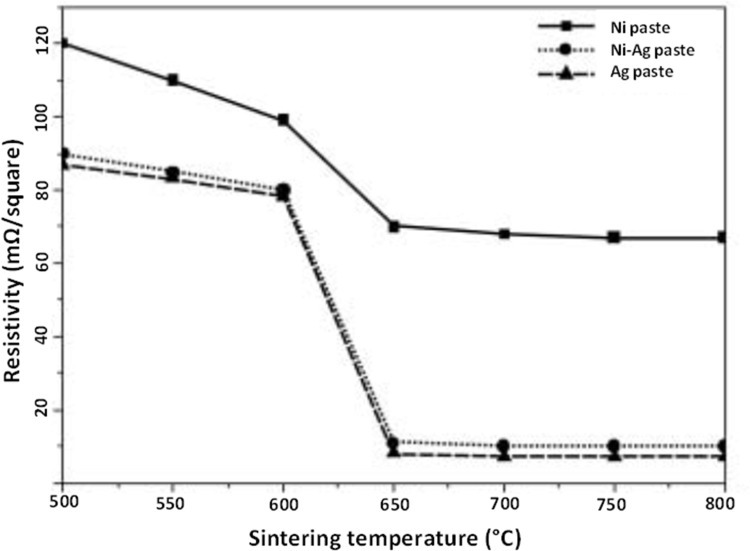
The effect of the sintering temperature on the resistivity of films based on nickel, silver, and nickel@silver core@shell NPs. Reprinted from Jing JJ, Xie J, Chen GY, Li WH, Zhang MM. Preparation of nickel-silver core-shell nanoparticles by liquid-phase reduction for use in conductive paste. J Exp Nanosci. 2015;10:1347–1356. Copyright 2015 Taylor and Francis.^55^

Jing et al^55^ films based on Ni@Ag NPs sintered at temperatures from 500°C to 800°C. Such coatings after sintering at 650°C had a sheet resistance of 11 mΩ/square. Therefore, the conductivities of films composed of Ni@Ag NPs were higher than those of the nickel paste, and similar to those of the silver paste, as is shown in Figure 10.

In the research presented in the paper,^22^ the dependence of the conductivity of hybrid ink coatings composed of Ni@Ag NPs and Ag NPs on their concentrations as well as sintering temperature was analyzed. It was noticed that by the doping of coatings mainly formed from nickel@silver nanoparticles at the average size of about 220 nm with smaller Ag NPs (~30 nm) the sintering temperature was decreased. Moreover, the modification of coatings mainly based on Ni@Ag NPs with Ag NPs affects their conductivity. The improvement of conductive properties of Ni@Ag NPs-based films, after they were doped with Ag NPs, at all ranges of applied sintering temperature was observed. However, at a lower sintering temperature from 130°C to 200°C, the highest effect of smaller Ag NPs addition was noticed.

**Figure 11:**
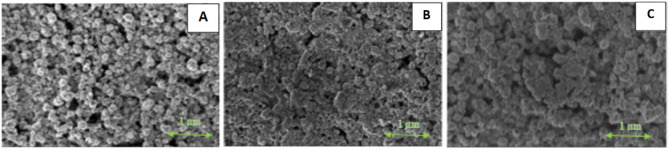
SEM images of Ni@Ag coatings: after drying (**A**); after drying, dipping in OA (1 wt%) and drying (40 °C) (**B**); after drying, dipping in OA (1%) followed by drying and sintering (100 °C, 30 min) (**C**). Reprinted from Pajor-Świerzy A, Pawłowski R, Sobik P, Kamyshny A, Szczepanowicz K. Effect of oxalic acid treatment on conductive coatings formed by Ni@Ag core–shell nanoparticles. Materials. 2022;15(1):305. © 2022 by the authors. Licensee MDPI, Basel, Switzerland. This article is an open access article distributed under the terms and conditions of the Creative Commons Attribution (CC BY) license.^49^

Following the global trend of producing flexible electronic devices and taking into account the economic considerations of the process of obtaining conductive paths and materials, the sintering methods that would enable obtaining coatings with good conductive properties while maintaining the low temperature of their fabrication were proposed. In this context, oxalic acid (OA), which is characterized by specific properties (antioxidative, reducing, and complexing for metallic ions as well as low decomposition temperature) was used as a “chemical sintering agent” for the fabrication of coatings based on Ni@Ag NPs.^49^ Such films were first treated with oxalic acid and then additionally heated at various temperature ranges. The treatment of coatings with OA at a concentration of 1% as an optimal one was selected due to the films with the lowest resistivities in the all range of applied sintering temperatures were obtained. Such an approach by using a “chemical sintering agent” allowed for decreasing the sintering temperature to 100°C, which can be applied for the sintering of coatings on heat-sensitive substrates like paper, polymers, or textiles. As is presented in Figure 11, the NPs in the coating treated with an optimal concentration of OA after the drying process (40°C for 15 min, Figure 11B) were much more connected in comparison to a coating not treated with oxalic acid (Figure 11A). Similarly, the good merging of Ni@Ag NPs in coatings dipped in solution of OA and then sintered at 100°C (Figure 11C) was observed, which resulted in a decrease in the resistivities of such films.

As an alternative approach to the thermal sintering method for the preparation of conductive structures based on Ni@Ag NPs UV-Vis irradiation process was used.^50^ The advantage of this process in comparison to the thermal sintering method is the lowering of risk of the destruction of heat-sensitive substrates due to performing the process without heating. The presented results in^50^ suggest that the sintering process by using the UV-Vis irradiation method is an effective approach for obtaining conductive coatings based on Ni@Ag NPs. The calculated conductivity was 29–30% in comparison to that for the bulk nickel. Therefore, the UV-Vis sintering process is a promising method for the future application of inks based on Ni@Ag NPs for the fabrication of electronic materials on flexible, heat-sensitive substrates. According to the application of Ni@Ag NPs for the fabrication of flexible electronics, Mohammadi et al^57^ showed the preparation process of inks based on the flame-synthesized nickel@silver nanopowders, which provided high conductivity without the sintering process.

**Figure 12:**
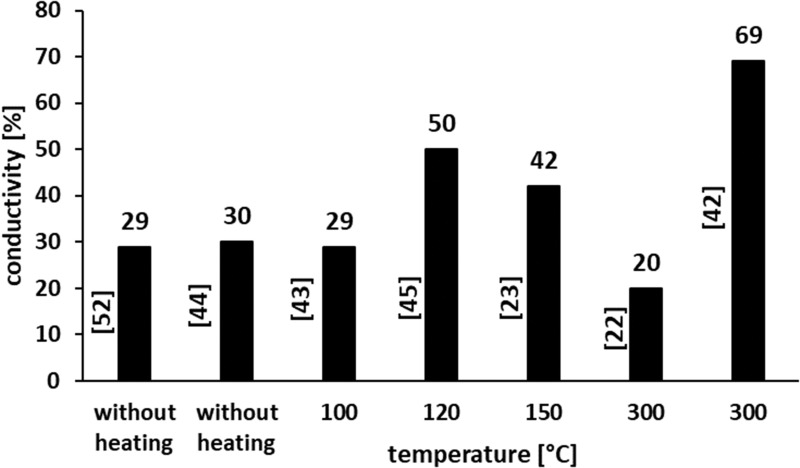
Summary of the results of coatings with the highest conductive properties obtained at the lowest sintering temperature (in square brackets [] the references are presented).

In Figure 12, the summary of the “most promising” results related to the highest conductive properties obtained for coatings at the lowest sintering temperature is presented. As is expected at the highest sintering temperature (300°C) the coatings with the highest conductivity of 69% (in comparison to that for bulk nickel) were formed.^48^ However, at a lower sintering temperature of 120°C also quite good conductivity of 50% of that for bulk nickel was obtained.^51^ The promising results were also obtained for coatings sintered without heating.^50^,^57^

## Outlook

The core@shell NPs composed of nickel (core) and silver (shell) as functional and active components of conductive materials (inks or pastes) were proposed as an alternative compound for costly silver or gold NPs. The rapid and noticeable progress in scientific knowledge and technological innovation in the fabrication of metallic Ni@Ag nanoparticles has been observed. Looking forward, there are many outlooks and future perspectives on bimetallic nanoparticles. However, the gap in the basic research on conductive materials composed of Ni@Ag NPs can be noticed. Also, converting scientific research into industrial technology applications is still a major challenge. Several problems need to be solved before the efficient implementation of such materials into large-scale industries. Firstly, more studies on the stability and shelf life of inks based on Ni@Ag NPs are needed. It is important for their future application in electronics fabrication to avoid the risk of decreasing or even losing their conductive properties. Moreover, more attention should be paid to up-scaling of the synthesis process of nanoparticles and the preparation of ink based on them, which is an important stage of the transfer of research from laboratory scale to industrial application. Such process can be limited by Ni@Ag NPs aggregation, contamination, degradation, or by economic aspects. It is obvious, that needing more amount of reagents generates higher costs of production. Besides, the gap in research on the long-term stability of the printed conductive materials based on Ni@Ag core@shell NPs under various environmental factors (temperature, humidity, oxygen, etc) was noticed. The analysis of their conductivity, adhesion to the substrate, morphological and topographical properties during the time will be useful for avoiding the process of their degradation. The improvement of low-temperature sintering methods compatible with heat-sensitive substrates that will be effectively working is also required. Focusing on a green chemistry approach and developing of sintering method friendly to the environment is also an important issue. The safety and regulation of Ni@Ag nanoparticles are important parameters that need to be considered for their future industrial application. Their impact on the environment is most likely unknown and remains a major concern. Overall, even more research on Ni@Ag NPs is still required; however, we believe that such nanoparticles show great potential in application in many areas of human life such as catalysis, sensors, paints, semiconductors, and optics. The continuation of research and collaboration between materials scientists, chemists, physics, and engineers will be necessary to fully realize the future prospective of the application of Ni@Ag bimetallic core@shell nanoparticles.
